# Motor Capabilities in Children with ADHD Are Improved after Brief Visuopostural Training

**DOI:** 10.3390/neurolint15030050

**Published:** 2023-06-28

**Authors:** Simona Caldani, Eric Acquaviva, Ana Moscoso, Benjamin Landman, Alexandre Michel, Richard Delorme, Maria Pia Bucci

**Affiliations:** 1 MoDyCo, UMR 7114 CNRS Université Paris Nanterre, 92001 Nanterre, France; simona.caldani@gmail.com; 2 Centre for the Functional Exploration of Balance in Children (EFEE), Robert Debré Hospital, 75019 Paris, France; 3 Child and Adolescent Psychiatry Department, Robert Debré Hospital, 75019 Paris, France; eric.acquaviva@gmail.com (E.A.); ana.moscoso@aphp.fr (A.M.); benjamin.landman@aphp.fr (B.L.); alexandre.michel@aphp.fr (A.M.); richard.delorme@aphp.fr (R.D.); 4 Paris University, 75005 Paris, France; 5 Human Genetics and Cognitive Functions, Institut Pasteur, 75015 Paris, France

**Keywords:** visuopostural training, posture, eye movements, children, ADHD

## Abstract

Children with ADHD show poor motor control. The aim of the present study was to test whether children with ADHD improved their motor performances (oculomotor as well as posture) after a short visuopostural training period. Two groups (G1 trained and G2 non-trained), each comprising 15 children with ADHD matched in IQ (intelligence quotient), sex, and age, participated in the study. Eye movements and postural sway were measured before (T1) and after (T2) 10 min of visuopostural training for the trained group and after 10 min of resting for the non-trained group. Training consisted of a visual search task performed while the child was standing on an unstable platform. At T1, oculomotor and postural abilities were statistically similar for both groups of children with ADHD (trained and non-trained). At T2, significant improvements in both oculomotor and postural capabilities were observed for the trained group but not for the non-trained group. These findings suggest that a short visuopostural training period could help children with ADHD to learn how to focus their visual attention in order to improve motor performance. Visuopostural training could allow a better integration of sensory inputs via central mechanisms, leading to improvement in both oculomotor and postural control. Further studies on a larger number of children with ADHD will be needed to confirm these findings and explore the eventual possible persistence of the training effect.

## 1. Introduction

Attention deficit/hyperactivity disorder (ADHD) is a neurodevelopmental disorder characterized by hyperactive behaviors and difficulties in controlling motor impulses and paying attention [1]. It affects up to 5% of children in the general population. Its association with frequent comorbidities leads to a major burden in childhood and later in adulthood [2]. Difficulties in both motor and inhibitory control have been found in children with ADHD [3]. Goulardins et al. [4] reported that motor deficiencies occur in 30–50% of children with ADHD. Our group [5,6,7], as well as other researchers [8,9,10], reported postural instability in ADHD children compared to control children, suggesting a deficit in sensory processing of the central structures responsible for body stability [11]. Smaller cerebellar volumes in subjects with ADHD have also been reported with a strong correlation between ADHD symptoms and the degree of reduction, suggesting a specific contribution of the cerebellum in the etiology of this disorder [12]. Indeed, abnormalities of the cerebellum have been reported in ADHD such as grey matter reductions in lobule IX and functional cerebellar dysconnectivity [13]. In an fMRI study, Kim et al. [14] hypothesized that the poor postural control of children with ADHD could be the consequence of decreased brain connectivity in the premotor cortex. The cerebellum is known to integrate different sensory inputs such as visual, vestibular, and somatosensory information responsible for postural control [15]. The cerebellum also plays a pivotal role in the realization of eye movements including the control of fixed eye movements through projections from the frontal eye field [16].

In the literature, poor eye movement performance has been reported in children with ADHD [17] when compared to typically developing children. Children with ADHD showed a larger number of errors during an anti-saccade task as well as frequent intrusive saccades during smooth pursuit and fixation, supporting the hypothesis that these difficulties could be explained by the presence of altered inhibitory mechanisms which are probably linked to a dysfunction of the central structures responsible for saccade triggering [5,18].

Several studies have explored whether training could improve motor and cognitive capabilities in subjects with ADHD. The meta-analysis by Cerrillo-Urbina et al. [19] reported eight studies with aerobic exercise interventions that improved attention, hyperactivity, impulsivity, anxiety, executive function, and social disorders in children with ADHD, most likely due to modification and regulation of the structures (prefrontal and cingulate cortex, brainstem, and cerebellum) responsible for cognitive functions. More recently, Moradi et al. [20] reported that balance training (for seven weeks) was able to improve postural control in children with ADHD, maybe due to an adaptive mechanism of the sensory process and cerebellar integration in this population. Interestingly, Shams et al. [21], in a large group of subjects with ADHD, pointed out the role of attention in maintaining postural control capabilities; the authors improved the postural control of these subjects by training their attention capabilities.

Furthermore, other researchers have explored the possibilities of improving cognitive abilities with eye movement exercises in children with ADHD. For instance, Janmohammadi et al. [22] tested the effects of an oculomotor intervention on inhibitory capabilities in a small group of children with ADHD (from 6–10 years old). The training lasted five weeks (two 30-min sessions per week) and was based on visual fixation and pursuit eye movements. The authors reported improvements in the inhibitory control system in children who benefited from the eye-tracking intervention, suggesting that oculomotor training could lead to an adaptive mechanism in the prefrontal/frontal cortex responsible for eye movements and inhibitory control. Lee et al. [23] also proposed a computerized eye-tracking training program for children with ADHD (aged between 6 and 12 years) consisting of several computer games in order to improve inhibitory control, mental flexibility, and attention. They reported improvements in latency and accuracy for both prosaccades as well as antisaccades, supporting the hypothesis that eye-tracker training could influence executive functions controlled by the frontal lobes. Recently, our group reported beneficial effects of a visuoattentional training on smooth pursuit performance in 8-year-old children with ADHD, showing that attentional mechanisms controlled by cortical structures could be improved by a short visuoattentional training period [24]. These findings were attributed to improvements in the prefrontal and parietal circuits. However, in this previous study, we explored the effects of a visuoattentional training on eye movement behavior only; in the present study, we explore whether a short visuopostural training period in which attentional and motor skills are solicited at the same time (dual task) could affect eye movement performance as well as postural ability in children with ADHD. Our hypothesis is that visuopostural training could allow children with ADHD to focus their attention, enabling a better use of sensory information and a better cerebellar integration, leading to an improvement in motor performances (oculomotor as well as postural activities).

## 2. Materials and Methods

### 2.1. Subjects

Two groups (trained and non-trained), each comprising 15 children with ADHD, age-, sex-, and IQ-matched, were included in the study. They were recruited at the Child and Adolescent Psychiatry Department, Robert Debré Hospital (Paris, France) (Table 1). All children were evaluated by trained child psychiatrists. The diagnosis of ADHD followed DSM-5 criteria [1]. The severity of the ADHD symptoms was estimated by the ADHD Rating Scale-parental report (ADHD-RS). All children with ADHD were also assessed using the Wechsler scale [25]. Note also that the children were naïve of any psychotropic drugs. The inclusion criteria were no history of vestibular, orthopedic, neurological, or psychiatric pathology; absence of drug use; normal corrected visual acuity (in each eye 20/25); normal mean intelligence quotient (IQ, evaluated with WISC-IV; between 80 and 115); and ADHD symptoms.

Exclusion criteria were any known neurological disorders, comorbidities, visual impairment, vestibular disorder, orthopedic disorder or surgeries and drug use. The inclusion of children was conducted by trained psychiatrists.

The assignment of a subject to a specific group (trained or non-trained group) was generated in an unpredictable random sequence (by computer program) and the sequence was implemented in a way that concealed the treatments (rest vs. training) until patients had been formally assigned to one of the two groups. After assignment to the training or non-training group, each child was given a code. The experimenter who analyzed the data was blinded to which group the child was in.

**Figure 1:**
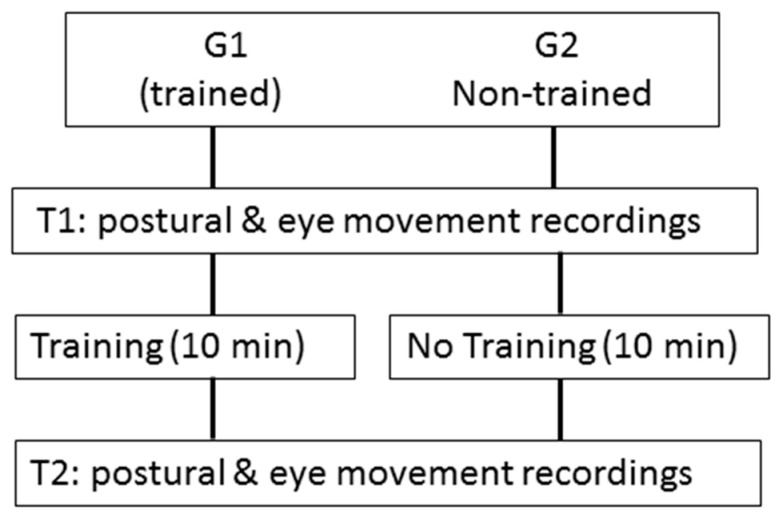
Description of trial design.

Eye movements and posture were recorded at two times, T1 and T2, respectively, i.e., before and after 10 min of visuopostural training for the trained group, and before and after 10 min of rest (i.e., without visuopostural training) for the non-trained group. During the 10 min of rest, children in the non-trained group were free to look around them as they sat on a chair speaking with one of the experimenters (see Figure 1). The investigation followed the principles of the Declaration of Helsinki and was approved by our Institutional Human Experimentation Committee (Comité de Protection des Personnes CPP Île-de-France). Written consent was obtained from the children and the children’s parents after the experimental procedure had been explained to them.

### 2.2. Eye Movement Recording

The Mobile EyeBrain Tracker (EBT), a non-invasive system, was used to record horizontal and vertical eye movements employing an infrared camera (recording frequency of 300 Hz). The precision of the mobile EBT is about 0.25 deg. Calibration under binocular viewing was performed before pursuit recording. The calibration procedure consisted in fixating a grid of 13 points (diameter 0.5 deg) mapping the screen (for more details, see our previous work [26]). The children were seated on a chair in a dark room, in front of a flat screen displaying the pursuit eye movements. The head of the child was held straight with a head-rest; viewing was binocular. After the calibration procedure, ocular motor tasks were presented to the subject. We recorded anti-saccades, pursuits and fixations (see below for details).

The pursuit task consisted of following a slowly moving visual target. The stimulus (a red circle of 0.5°) was moved at a constant velocity (15°/s) on a 22-inch computer monitor. The target was initially placed in the central position (0 deg) and then moved horizontally to one side until it reached the ±20 deg location, where it reversed abruptly and moved to the opposite side. A total of nine cycles were run and included in the analysis. The children were asked to follow the target with their eyes (for more detail, see our previous study [26]).

In the fixation task, all children performed two visual conditions. In the first one, named simple fixation, the child was asked to fix their gaze on the target appearing in the center (filled white circle subtending a visual angle of 0.5°) of the black screen during 30 s. In the second one, named fixation with distractors, the child had to maintain fixation on the central target and to inhibit saccades toward the distractors. The distractor was a white smile target (of 0.5°) appearing for a random duration from 500 to 2000 ms and calling for horizontal saccade amplitudes from 5° to 20°. The distractor was presented during the fixation with distractor (30 s); in total, 8 distractors were presented during each fixation with distractor trial. Instructions were given to the child to try to fixate the central target as well as possible (in the simple fixation task) and to try to fixate the central target as well as possible without looking at the distractors (in the fixation with distractor task). Each child performed two blocks of different types of eye movements and each block was separated by a few minutes of rest. In other words, each child performed the pursuit task twice, the simple fixation task twice, and the fixation with distractor task twice.

### 2.3. Postural Recording

To evaluate postural control the Multitest Equilibre system (www.framiral.fr (accessed on 11 May 2023)) was used [6,27]. In a dark room, the child had to stand upright on the platform with their arms along the side of the body and the feet position standardized on footprints (distance and angle between heels: 11 cm and 30°, respectively). The surface of the center of pressure displacement (CoP) was measured on the unstable platform and under two different viewing conditions: eyes open (EO), fixating a target at a distance of 250 cm (target projected on a screen in front of the children at their eye level), and eyes closed (EC). Each of the two postural recordings was performed for 30 sec and the order of the conditions (EO and EC) was randomized. The displacement of the Center of Pressure (CoP) was sampled at 40 Hz and digitized with 16-bit precision. Postural recording was performed in the unstable platform condition, i.e., dynamic sway-referenced conditions in which the platform could move in all axes, in a free-floating manner, in reference to the child’s sway directions. In the referenced dynamic sway conditions, the platform itself moves in the same direction as the pressure exerted by the subject’s feet. As a result, the unstable platform dramatically decreases the ability of the plantar sole’s exteroceptors to discriminate against the pressure and shear exerted by the child on the ground and thereby increases the demand on the child’s visual and vestibular systems to maintain balance. For each child, two postural recordings were conducted in each visual condition (EO and EC).

Both eye movements and postural tests were conducted at two times, T1 and T2, respectively, i.e., before and after 10 min of visuopostural control training for the subjects allocated to the trained group, and before and after 10 min of rest (i.e., without any training) for the non-trained group.

### 2.4. Visuopostural Training

**Figure 2:**
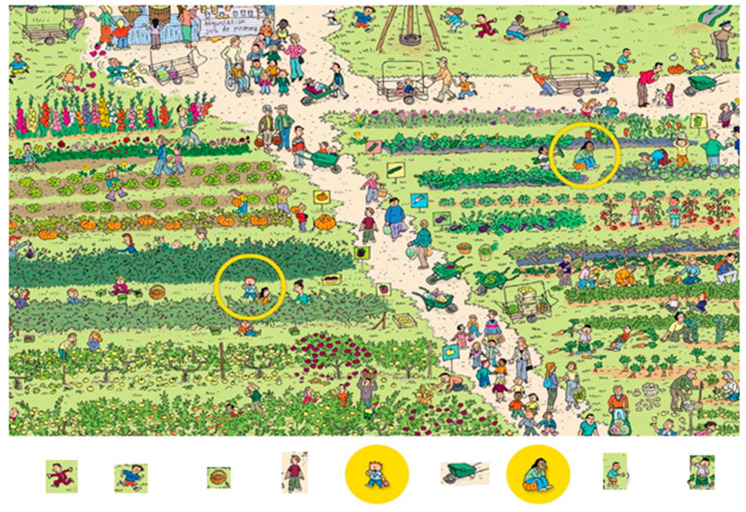
Example of an image used to induce visual search during the training period. The child is asked to search for the object/person shown below the image and to point to it with a laser pointer. In this example the first person to search for was a boy dressed in red, then the boy with the blue t-shirt, and so on until the child had found all the people shown below the image. The persons in yellow circles were used at the beginning of the training to ensure that the child had understood the task correctly. After this scene, other scenes were presented, and the child had to conduct an image/person search for 10 min.

The child was on an unstable platform of Framiral, which moved at the same time as the child was asked to look in front of him/her at a screen (2.5 m away), on which different types of images were projected. The children were asked to look at an image projected in a circle (visual angle: 21°) and they also had to locate the position of several objects/persons in the scene (seven objects/persons for each scene, see Figure 2). The object/person to be located was shown in an insert (visual angle: 2°) below the visual scene. The child had to point with a laser-pointer at the object/person when he/she saw it. The object/person was of different colors. The search task was quite difficult in order to challenge the visual attention demand of children with ADHD. Note that the length of time during which each scene was presented differed depending on the visual search ability of each child. Consequently, several scenes were presented to the child in order to make a visuopostural training of 10 min for each child. Note also that we decided to test the effects of a shorter training course in order to avoid the effects of fatigue since the children with ADHD reported difficulties in focusing their attention for a long time.

### 2.5. Data Analysis

The MeyeAnalysis software was used to determine automatically the onset and the end of each saccade by using a ‘built-in saccade detection algorithm’. All detected saccades were verified by the investigator.

Concerning the pursuit task, catch-up saccades were defined as saccades in the target direction that served to reduce position error and to bring the eye closer to the target. The number of catch-up saccades was counted for each trial. Amplitude was measured for each catch-up saccade. Pursuit gain corresponds to the relationship between the speed of the eye and the speed of the target, and it was obtained by dividing eye velocity by target velocity for each trial. If its value is around 1 it means that the correspondence between the target and the eye is perfect. Scores were then averaged across trials for each test [26]. Concerning fixation tasks, for both visual conditions (simple fixation and fixation with distractors) we counted the number of saccades made during the task [28].

Postural control performance was evaluated using the surface area of the CoP (in cm^2^), and the mean velocity (mm/s). The surface of the CoP was calculated corresponding to the area of an ellipse encompassing 90% of all CoP data point excursions. A larger surface area of the CoP means poor postural control. Mean velocity is an efficient indicator to quantify the neuromuscular activity required to regulate postural control [29]: high values of mean velocity correspond to a strong muscular effort to maintain postural control.

### 2.6. Statistical Analysis

Student’s *t* test was performed in order to compare the age, IQ, and ADHD-RS score in the two groups of children (trained and non-trained). A repeated measures ANOVA was run between the two groups of children (trained and non-trained) as between-subjects factor on each oculomotor variable (number of saccades reported during pursuits and number of saccades measured in simple fixations and fixations with distractors) recorded two times condition (2 levels: T1 and T2) as a within-subjects factor. A repeated-measures ANOVA was also run between the two groups of children (trained and non-trained) as a between-subjects factor on each postural parameter (the surface area and mean velocity of the CoP), recorded in two time conditions (2 levels: T1 and T2), and under two distinct visual conditions (2 levels: EO and EC) as a within-subjects factor. Post hoc comparisons were made with the Bonferroni test. Significance was considered when the *p*-value was below 0.05. All statistical analyses were processed using JASP software (a free, open-source program for statistical analysis supported by the University of Amsterdam).

## 3. Results

Table 1 shows the clinical characteristics of the two groups of children (trained and non-trained). Student‘s *t* test failed to report any statistical difference between G1 (trained) and G2 (non-trained).

### 3.1. Eye Movements

**Figure 3:**
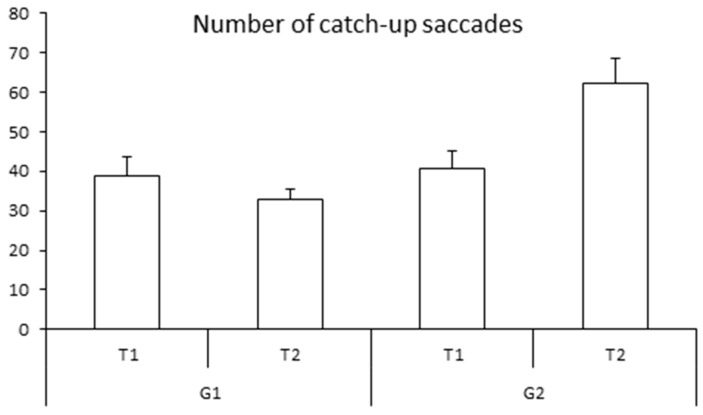
Means and standard deviations of the number of catch-up saccades during pursuits at T1 and T2 for both groups of children (G1 and G2).

Figure 3 shows the mean number of catch-up saccades for both groups of children examined at T1 and T2, respectively. The ANOVA revealed a significant group effect (F_(1,28)_ = 6.51, *p <* 0.01 η^2^ = 0.12): the number of catch-up saccades was smaller in the trained group than in the non-trained group. The ANOVA also revealed a significant interaction between time and group of children (F_(1,28)_ = 11.80, *p* < 0.002 η^2^ = 0.10). The Bonferroni post hoc test showed that for the trained group only (G1), the number of catch-up saccades decreased significantly at T2 (*p* < 0.01). The number of catch-up saccades was similar in both groups at T1 (*p* > 1). The number of catch-up saccades in the G2 (non-trained group) failed to show a significant difference (*p* = 0.2, see Table 2).

The ANOVA failed to show any significant group and time effect for pursuit gain. The mean gain was at T1 0.87 ± 0.03 and 0.86 ± 0.03 for the G1 and G2 (trained and non-trained group, respectively) and at T2 0.96 ± 0.03 and 0.85 ± 0.03, for the G1 and G2 (trained and non-trained group, respectively).

**Figure 4:**
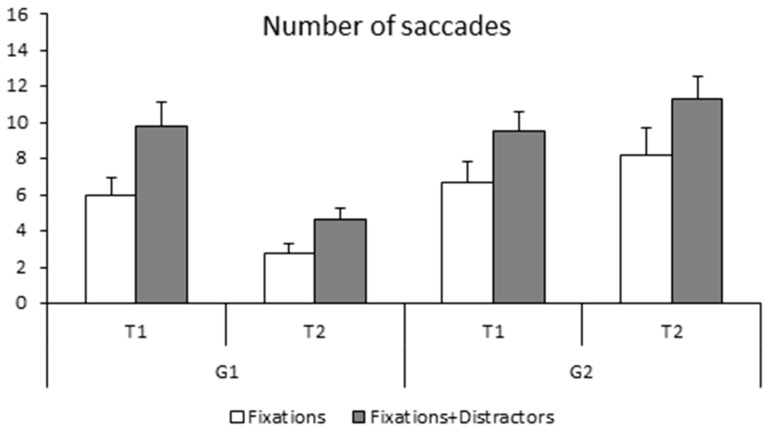
Means and standard deviations of the number of saccades during simple fixation and fixation with distractor at T1 and T2 for both groups of children (G1 and G2).

The number of saccades made during the fixation task (simple and with distractors) is shown in Figure 4. The ANOVA reported a significant group effect (F_(1,28)_ = 5.43, *p* < 0.02 η^2^ = 0.01); the non-trained group made significantly more saccades during fixation tasks with respect to the trained group. The ANOVA also reported a time effect (F_(1,28)_ = 46.09, *p* < 0.001 η^2^ = 0.01), as the number of saccades was significantly smaller at T2 than at T1. There was also a significant task effect also (F_(1,28)_ = 11.98, *p* < 0.002 η^2^ = 0.07), with saccades occurring more frequently in the fixation with distractors task. Lastly, the ANOVA showed a significant interaction between the groups and time (F_(1,28)_ = 46.09, *p* < 0.001 η^2^ = 0.07). Bonferroni post hoc tests showed that the number of saccades made by the trained group was significantly smaller at T2 with respect to T1 (*p* < 0.001) while the number of saccades made at T1 was similar in the two groups of children (trained and non-trained group, *p* > 1). The number of saccades in the non-trained group did not change at T2 with respect to T1 (*p* < 0.9).

### 3.2. Postural Control

**Figure 5:**
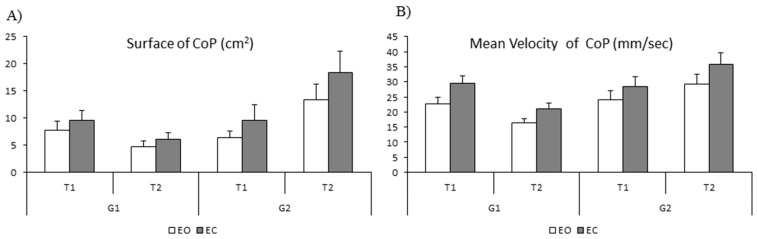
Means and standard deviations of the surface in cm^2^ (**A**) and mean velocity in mm/s of the CoP (**B**) in eyes open (EO) and eyes closed (EC) condition at T1 and T2 for both groups of children (G1 and G2).

The mean values of the surface (cm^2^) and the mean velocity of the CoP (mm/sec) measured with eyes open (EO) and eyes closed (EC) are shown in Figure 5 (A and B, respectively). The ANOVA reported a significant effect of vision (F_(1,28)_ = 5.92, *p* < 0.02 η^2^ = 0.02); the surface of the CoP was significantly larger with eyes closed than with eyes open. The ANOVA also showed a significant interaction between time and group (F_(1,28)_ = 22.03 *p* < 0.001 η^2^ = 0.08). Bonferroni post hoc tests revealed that the surface of the CoP of the trained group was significantly smaller at T2 with respect to T1 (*p* < 0.04), while at T1 it was similar in the two groups of children (trained and non-trained group, *p* > 1). For the non-trained group, the mean surface area of the CoP increased significantly at T2 with respect to T1 (*p* < 0.04).

Lastly, for the mean velocity of the CoP, the ANOVA reported a significant effect of vision (F_(1,28)_ = 4.66 *p* < 0.04 η^2^ = 0.03), showing a smaller mean velocity in the eyes-open condition and a significant interaction between time and group (F_(1,28)_ = 22.26 *p* < 0.001 η^2^ = 0.07). The Bonferroni post hoc test revealed that the mean velocity of the CoP of the trained group was significantly smaller at T2 with respect to T1 (*p* < 0.001), while the surface of the CoP at T1 was similar in the two groups (trained and non-trained, *p* > 1). For the non-trained group, the mean velocity was similar at T1 and T2 (*p* > 0.13).

## 4. Discussion

The present study aimed to explore whether a visuopostural training of 10 min could improve general motor performance in children with ADHD. More specifically, we examined oculomotor and postural performance, which are controlled by corticocerebellar networks that are known to be deficient in children with ADHD (see Introduction Section). To our knowledge, this study is the first to show an improvement in general motor function at the level of both ocular and body control in children with ADHD after a short training period in which children used simultaneously different sensory inputs: (i) vision input to search for a small target in a picture; (ii) proprioception and vestibular inputs to avoid falling and to keep the body upright. As previously discussed, we know that a long [22] or a short [24] oculomotor training period could improve cognitive capabilities in children with ADHD by helping them to better focus their attention and better control their impulsivity. In our study, attention was trained together with vision, because the children were asked to focus their attention in order to accomplish the search task correctly. Because eye movement control is related to attention and cognitive capabilities [30,31], the improvement in oculomotor performance observed for pursuit and fixation tasks could be due to a better use of visual attention. At the same time, the children were trained to control their body stability as they were on an unstable platform. This short training period was able to improve their postural control, most likely due to a better use of sensory inputs responsible for balance control.

In the present study, children were trained by using a dual task (searching for targets on the images and controlling their postural sway), which is a simplification of ecological settings, where the balance task is usually associated with other cognitive tasks (e.g., talking while listening to music, etc.). In this dual task situation, the contribution of structures involved in motor attention and internal representation is increased, and attentional resources must be allocated to perform both tasks correctly. Attention is distributed between the two simultaneous tasks in various ways according to the strategy chosen by the child, and the performance of the two tasks depends on how much attention they require. The performance of a dual postural task in children with ADHD has been explored and several studies reported that a secondary task needing attention improved attentive performance in these children [32,33,34]. We suggest that this type of training—in which the child had to perform simultaneously a vision and a postural task, which is more demanding in terms of attention resources—could be beneficial for children with ADHD by helping them to learn how to better focus their attention in order to succeed in each task.

It is well known that the cerebellum plays a major role not only in motor control but also in cognition (see the review [35]), and it could be considered the main center for the integration and connection of neural circuits subserving the behavior of subjects across all domains. Gottwald et al. [36], for instance, demonstrated that focal cerebellar lesions lead to impairment in higher cognitive processes and attention. Craig et al. [37] showed that lesions to Crus II of the left posterior cerebellum impaired performance in visual attention. The implication of the cerebellum in subjects with ADHD was reviewed by Phillips et al. [38], who reported several studies showing cerebellar changes in ADHD. In subjects with ADHD, Rosenberg et al. [39] observed that functional connectivity in brain regions implicated in attentional capacity are disrupted and that ADHD is associated with altered functional connectivity within and between the cerebellar and frontoparietal networks [40,41]. Based on these studies, it seems reasonable to assume that cerebellar activity could be improved by visuopostural training, and that the cerebellar networks related to visual attention may be more functional after such a type of training. We suggest that children with ADHD could take advantage of training in which dual-task protocols are involved in order to reinforce their attention capabilities. A dual-task training type could be an easy training model to be used by children to improve their sensorial inputs in a simple way; clinicians could also take advantage of this type of training for the better care of children with ADHD. Recall that the motivation and involvement of children during training are very important to achieving a behavioral improvement in these kinds of children.

Finally, we hypothesize that such training allowed the children to better integrate sensory information via the cerebellum. In other words, the improvement in the motor control observed in children with ADHD could be due to central plasticity. In young subjects, postural training could increase grey matter and cortical structures [42]. Furthermore, the group of Hoekzema [43,44] reported that a cognitive training model (based on working memory, cognitive flexibility, and attention) induced in children with ADHD a significant increase in gray matter in the temporal, prefrontal, and frontal cortex, as well as the right inferior–posterior cerebellum. They suggested that such cognitive training could lead to synaptic adjustments in the front-cerebellar networks, similarly to the effect of psychostimulant medications. Further studies combining training programs and imaging techniques are needed to understand the plasticity of the human brain.

## 5. Limitations

Despite the encouraging results, there are some limitations of this study. First, these results need to be confirmed via the testing of a larger group of children with and without ADHD; more importantly, the present study measured only the immediate effect of visuopostural training; it is unknown whether the improvement persisted after the training. Consequently, follow-up studies are needed to explore further the sustainability of the training effects. Second, the underlying neural mechanism responsible for such motor improvement is still unknown; in the Discussion Section, we proposed some hypotheses that need to be confirmed via neurophysiological measures.

## 6. Conclusions

To summarize our findings, we reported a beneficial effect of a short visuopostural training model with respect to motor control (eye movements as well as postural stability) in children with ADHD. This improvement could be due to a better use of attentional resources in visual and postural activities, most likely due to the adaptive mechanisms of the nervous system.

## Figures and Tables

**Table 1:** Clinical characteristics of the two groups of children with ADHD (trained and non-trained) tested. Mean and standard deviations of age (years), IQ, ADHD-RS score.

	G1 Trained Children (N =15)	G2 Non-Trained Children (N = 15)
Boys/girls	12/3	11/4
Age (years)	8.8 ± 0.4	8.6 ± 0.3
IQ (WISC-IV)	100 ± 5.0	102 ± 4.0
ADHD-RS score	40 ± 2.0	38 ± 2.1
Inattention	20 ± 1.1	18 ± 2.2
Hyperactivity	21 ± 2.0	20 ± 1.9

**Table 2:** Means and standard deviations of the pursuit gain at T1 and T2 for both groups of children (G1 and G2).

	T1 Pursuit Gain	T2 Pursuit Gain
**G1** **(trained children)**	0.83 ± 0.03	0.86 ± 0.03
**G2** **(non-trained children)**	0.86 ± 0.03	0.85 ± 0.03

## Data Availability

The data presented in this study are available on request from the corresponding author.

## References

[1] American Psychiatric Association APA (2022). Diagnostic and Statistical Manual of Mental Disorders.

[2] Weissenberger S., Ptacek R., Klicperova-Baker M., Erman A., Schonova K., Raboch J., Goetz M. (2017). ADHD, Lifestyles and Comorbidities: A Call for an Holistic Perspective—From Medical to Societal Intervening Factors. Front. Psychol..

[3] Lachambre C., Proteau-Lemieux M., Lepage J.F., Bussières E.L., Lippé S. (2021). Attentional and executive functions in children and adolescents with developmental coordination disorder and the influence of comorbid disorders: A systematic review of the literature. PLoS ONE.

[4] Goulardins J.B., Marques J.C., De Oliveira J.A. (2017). Attention deficit hyperactivity disorder and motor impairment. Percept. Mot. Skills.

[5] Bucci M.P., Seassau M., Larger S., Bui-Quoc E., Gerard C.L. (2014). Effect of visual attention on postural control in children with attention deficit/hyperactivity disorder. Res. Dev. Disabil..

[6] Bucci M.P., Stordeur C., Acquaviva E., Peyre H., Delorme R. (2016). Postural instability in children with ADHD is improved by methylphenidate. Front. Child Neurodev. Psychiatry.

[7] Caldani S., Razuk M., Septier M., Barela J.A., Delorme R., Acquaviva E., Bucci M.P. (2019). The Effect of Dual Task on Attentional Performance in Children With ADHD. Front. Integr. Neurosci..

[8] Wang J., Wang Y., Ren Y. (2003). A case control study on balance function of attention deficit hyperactivity disorder (ADHD) children. J. Peking Univ. Health Sci..

[9] Zang Y., Gu B., Qian Q., Wang Y. (2002). Objective measurement of the balance dysfunction in attention deficit hyperactivity disorder children. Chin. J. Clin. Med..

[10] Zoccante L., Ciceri M.L., Chamitava L., Di Gennaro G., Cazzoletti L., Zanolin M.E., Darra F., Colizzi M. (2021). Postural Control in Childhood: Investigating the Neurodevelopmental Gradient Hypothesis. Int. J. Environ. Res. Public Health.

[11] Buderath P., Gartner K., Frings M., Christiansen H., Schoch B., Konczak J., Gizewski E.R., Hebebrand J., Timmann D. (2009). Postural and gait performance in children with attention deficit/hyperactivity disorder. Gait Posture.

[12] Stoodley C.J. (2016). The cerebellum and neurodevelopmental disorders. Cerebellum.

[13] Tomasi D., Volkow N.D. (2012). Abnormal functional connectivity in children with attention-deficit/hyperactivity disorder. Biol. Psychiatry.

[14] Kim S.M., Hyun G.J., Jung T.W., Son Y.D., Cho I.H., Kee B.S., Han D.H. (2017). Balance Deficit and Brain Connectivity in Children with Attention-Deficit/Hyperactivity Disorder. Psychiatry Investig..

[15] Horak F.B. (2006). Postural orientation and equilibrium: What do we need to know about neural control of balance to prevent falls?. Age Ageing..

[16] Krauzlis R.J., Goffart L., Hafed Z.M. (2017). Neuronal control of fixation and fixational eye movements. Philos. Trans. R Soc. Biol. Sci..

[17] Goto Y., Hatakeyama K., Kitama T., Sato Y., Kanemura H., Aoyagi K., Sugita K., Aihara M. (2010). Saccade eye movements as a quantitative measure of frontostriatal network in children with ADHD. Brain Dev..

[18] Caldani S., Isel F., Septier M., Acquaviva E., Delorme R., Bucci M.P. (2020). Impairment in attention focus during the Posner cognitive task in children with ADHD: An eye tracker study. Front. Pediatr. Sect. Child Adolesc. Psychiatry.

[19] Cerrillo-Urbina A.J., García-Hermoso A., Sánchez-López M., Pardo-Guijarro M.J., Santos Gómez J.L., Martínez-Vizcaíno V. (2015). The effects of physical exercise in children with attention deficit hyperactivity disorder: A systematic review and meta-analysis of randomized control trials. Child Care Health Dev..

[20] Moradi J., Jalali S., Bucci M.P. (2020). Effects of Balance Training on Postural Control of Children with Attention Deficit/Hyperactivity Disorder. Iran. J. Pediatr..

[21] Shams A., Dehkordi P.S., Tahmasbi F., Sangari M. (2020). Are attentional instruction and feedback type affect on learning of postural and supra-postural tasks?. Neurol Sci..

[22] Janmohammadi S., Haghgoo H.A., Farahbod M., Overton P.G., Pishyareh E. (2020). Effect of a visual tracking intervention on attention and behavior of children with Attention Deficit Hyperactivity Disorder. J. Eye Mov. Res..

[23] Lee T.L., Yeung M.K., Sze S.L., Chan A.S. (2020). Computerized Eye-Tracking Training Improves the Saccadic Eye Movements of Children with Attention-Deficit/Hyperactivity Disorder. Brain Sci..

[24] Caldani S., Delorme R., Moscoso A., Septier M., Acquaviva E., Bucci M.P. (2020). Improvement of pursuit eye movement deficiency after short visuo-attentional training in ADHD. Brain Sci..

[25] Wechsler D. (2003). Wechsler Intelligence Scale for Children.

[26] Lions C., Bui-Quoc E., Wiener-Vacher S., Seassau M., Bucci M.P. (2013). Smooth pursuit eye movements in children with strabismus and in children with vergence deficits. PLoS ONE.

[27] Goulème N., Gérard C.L., Bucci M.P. (2015). Training effect on postural control in dyslexic children. PLoS ONE.

[28] Tiadi A., Gérard C.L., Peyre H., Bui-Quoc E., Bucci M.P. (2016). Immaturity of visual fixations in dyslexic children. Front. Hum. Neurosci..

[29] Geurts A.C., Nienhuis B., Mulder T.W. (1993). Intrasubject variability of selected force-platform parameters in the quantification of postural control. Arch. Phys. Med. Rehabil..

[30] Theewes J., Belopolsky A., Olivers C.N.L. (2009). Interactions between working memory, attention and eye movements. Acta Psychol..

[31] Van der Stigchel S., Meeter M., Theeuwes J. (2006). Eye movement trajectories and what they tell us. Neurosci. Biobehav. Rev..

[32] Shorer Z., Becker B., Jacobi-Polishook T., Oddsson L., Melzer I. (2012). Postural control among children with and without attention deficit hyperactivity disorder in single and dual conditions. Eur. J. Pediatr..

[33] Manicolo O., Grob A., Hagmannvon Arx P. (2017). Gait in children with attention-deficit hyperactivity disorder in a dual-task paradigm. Front. Psychol..

[34] Möhring W., Klupp S., Grob A. (2018). Effects of dual tasking and methylphenidate on gait in children with attention deficit hyperactivity disorder. Hum. Mov. Sci..

[35] Schmahmann J.D. (2019). The cerebellum and cognition. Neurosci Lett..

[36] Gottwald B., Mihajlovic Z., Wilde B., Mehdorn H.M. (2003). Does the cerebellum contribute to specific aspects of attention?. Neuropsychologia.

[37] Craig B.T., Morrill A., Anderson B., Danckert J., Striemer C.L. (2021). Cerebellar lesions disrupt spatial and temporal visual attention. Cortex..

[38] Phillips J.R., Hewedi D.H., Eissa A.M., Moustafa A.A. (2015). The cerebellum and psychiatric disorders. Front. Public Health.

[39] Rosenberg M.D., Finn E.S., Scheinost D., Papademetris X., Shen X., Constable R.T., Chun M.M. (2015). A neuromarker of sustained attention from whole-brain functional connectivity. Nat. Neurosci..

[40] Castellanos F.X., Margulies D.S., Kelly C., Uddin L.Q., Ghaffari M., Kirsch A., Shaw D., Shehzad Z., Di Martino A., Biswal B. (2008). Cingulate-precuneus interactions: A new locus of dysfunction in adult attention-deficit/hyperactivity disorder. Biol. Psychiatr..

[41] Barber A.D., Jacobson L.A., Wexler J.L., Nebel M.B., Caffo B.S., Pekar J.J., Mostofsky S.H. (2015). Connectivity supporting attention in children with attention deficit hyperactivity disorder. Neurol. Clin..

[42] Taubert M., Draganski B., Anwander A., Müller K., Horstmann A., Villringer A., Ragert P. (2010). Dynamic properties of human brain structure: Learning-related changes in cortical areas and associated fiber connections. J. Neurosci..

[43] Hoekzema E., Carmona S., Tremols V., Gispert J.D., Guitart M., Fauquet J., Rovira M., Bielsa A., Soliva J.C., Tomas X. (2010). Enhanced neural activity in frontal and cerebellar circuits aftercognitive training in children with attention-deficit/hyperactivity disorder. Hum. Brain Mapp..

[44] Hoekzema E., Carmona S., Ramos-Quiroga J.A., Barba E., Bielsa A., Tremols V., Rovira M., Soliva J.C., Casas M., Bulbena A. (2011). Training-induced neuroanatomical plasticity in ADHD: A tensor-based morphometric study. Hum. Brain Mapp..

